# Area under the curve of methotrexate and creatinine clearance are outcome-determining factors in primary CNS lymphomas

**DOI:** 10.1038/sj.bjc.6601472

**Published:** 2004-01-20

**Authors:** A J M Ferreri, E Guerra, M Regazzi, F Pasini, A Ambrosetti, A Pivnik, A Gubkin, A Calderoni, M Spina, A Brandes, F Ferrarese, A Rognone, S Govi, S Dell'Oro, M Locatelli, E Villa, M Reni

**Affiliations:** 1 Department of Radiochemotherapy, San Raffaele H Scientific Institute, Via Olgettina 60, Milan 20132, Italy; 2 Laboratorio di Standardizzazione per la Chimica Clinica, San Raffaele H Scientific Institute, Milan, Italy; 3 Department of Pharmacology, I.R.C.C.S. Policlinico San Matteo, University of Pavia, Italy; 4 Divisione Clinicizzata di Oncologia Medica, Ospedale Civile Maggiore, Verona, Italy; 5 Divisione di Ematologia, Policlinico G. B. Rossi, Verona, Italy; 6 Hematological Center of Russian Academy of Medical Sciences, Hematology and Intensive Care Department, Moscow, Russia; 7 Institut für Medizinische Onkologie Inselspital, Bern, Switzerland; 8 Divisione di Oncologia Medica ‘A’, Centro di Riferimento Oncologico, Istituto Nazionale Tumori, Aviano, Italy; 9 Department of Medical Oncology, Azienda Ospedale-Università, Padova, Italy; 10 Divisione di Radioterapia, Ospedale Regionale di Treviso, Treviso, Italy

**Keywords:** primary central nervous system lymphoma, methotrexate, plasmatic clearance, dose intensity, area under the curve, chemotherapy

## Abstract

Although high-dose methotrexate (HD-MTX) is the most effective drug against primary CNS lymphomas (PCNSL), outcome-determining variables related to its administration schedule have not been defined. The impact on toxicity and outcome of the area under the curve (AUC_MTX_), dose intensity (DI_MTX_) and infusion rate (IR_MTX_) of MTX and plasmatic creatinine clearance (CL_crea_) was investigated in a retrospective series of 45 PCNSL patients treated with three different HD-MTX-based combinations. Anticonvulsants were administered in 31 pts (69%). Age >60 years, anticonvulsant therapy, slow IR_MTX_ (⩽800 mgm^−2^h^−1^), and reduced DI_MTX_ (⩽1000 mgm^−2^wk^−1^) were significantly correlated with low AUC_MTX_ values. Seven patients (16%) experienced severe toxicity, which was independently associated with slow CL_crea_. A total of 18 (40%) patients achieved complete remission after chemotherapy, which was independently associated with slow CL_crea_. In all, 22 patients were alive at a median follow-up of 31 months, with a 3-year OS of 40±9%; slow CL_crea_ and AUC_MTX_ >1100 *μ*mol hl^−1^ were independently associated with a better survival. Slow CL_crea_ and high AUC_MTX_ are favourable outcome-determining factors in PCNSL, while slow CL_crea_ is significantly related to higher toxicity. AUC_MTX_ significantly correlates with age, anticonvulsant therapy, IR_MTX_, and DI_MTX_. These findings, which seem to support the choice of an MTX dose ⩾3 gm^−2^ in a 4–6-h infusion, every 3–4 weeks, deserve to be assessed prospectively in future trials. MTX dose adjustments in patients with fast CL_crea_ should be investigated.

High-dose methotrexate (HD-MTX) is the most effective drug against primary central nervous system lymphomas (PCNSL) (Reni *et al*, 1997; Ferreri *et al*, 2002b). Any chemotherapy regimen without HD-MTX is associated with outcomes no better than those obtained with radiotherapy (RT) alone in these malignancies (Schultz *et al*, 1996; O’Neill *et al*, 1999; Mead *et al*, 2000), while the survival benefit of the addition of other drugs to HD-MTX remains a matter of debate (Reni *et al*, 2001; Ferreri *et al*, 2002b). In spite of its central role in PCNSL treatment, the optimal dose, administration schedule, and dose timing of MTX have not been clearly defined. Moreover, contrary to what is observed in other malignancies in which MTX plays a crucial role, such as acute leukaemia (Evans *et al*, 1986) and osteosarcoma (Graf *et al*, 1994; Delepine *et al*, 1995; Bacci *et al*, 1998), where a significant association between MTX parameters and outcome has been reported, the impact on toxicity and outcome of MTX administration schedule has not been investigated in PCNSL.

The analysis of some MTX parameters, such as the area under the curve (AUC_MTX_), dose, dose intensity (DI_MTX_), and infusion rate (IR_MTX_), as well as the plasmatic creatinine clearance (CL_crea_) could allow us to identify subgroups of patients with increased risk of severe toxicity or disappointing outcome, as well as to define the optimal MTX administration schedule against PCNSL. This paper reports the analysis of the impact on toxicity and outcome of the above-mentioned variables in a retrospective multicentre series of 45 immunocompetent patients with PCNSL treated with HD-MTX-based primary chemotherapy.

## PATIENTS AND METHODS

### Study population

A questionnaire requesting epidemiological, clinical, histopathological, therapeutic, and survival data of immunocompetent patients with PCNSL treated with primary chemotherapy containing HD-MTX (⩾1 gm^−2^), followed or not followed by RT, was sent to the seven participating centres. In particular, the questionnaire included body weight and body surface area, creatinine clearance, theoretical and really administrated dose of MTX, duration of infusion, timing dose, and MTX serum levels at 0, 24, 48, and 72 h. The use of anticonvulsants, type and dose, was also analysed considering the capacity of some of these drugs to interfere with MTX metabolism (Jacobs *et al*, 1976). This study conformed to the tenets of the Declaration of Helsinki and all the patients accessioned provided signed informed consent to the treatment. This consent extended to the use of biological, histopathological, radiological, biochemical, and clinical data for scientific purposes.

### MTX variables

CL_crea_ value, determined before the start of chemotherapy, was obtained by the formula of Cockcroft and Gault (1976):


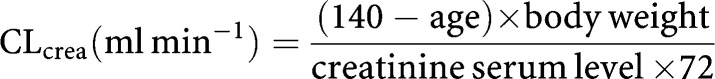


CL_crea_ value in females was considered as 85% of the value for males.

The MTX variables investigated were AUC_MTX_, dose, DI_MTX_, and IR_MTX_. The individual AUC_MTX_ (*μ*mol hl^−1^) related to the first course of chemotherapy was determined according to a one-compartment model by using the statistical population pharmacokinetic program P-PHARM-Version 3 (InnaPhase, 77420 Champs-sur-Marne, France), considering MTX dosage and serum levels at 0, 24, 48, and 72 h after drug infusion for calculation. DI_MTX_, expressed as mgm^−2^wk^−1^, was calculated by the Hryniuk method (Hryniuk and Goodyear, 1990). This was a ratio between the total dose of MTX administered (mgm^−2^) and the treatment duration expressed in days divided by 7. Treatment duration was calculated from the first day of the first course to the 22nd or 29th day of the last course (respectively for regimens administered every 3 or 4 weeks) (Hryniuk and Goodyear, 1990). The IR_MTX_, expressed as mgm^−2^h^−1^, was defined as the MTX dose (mgm^−2^) administered per hour during the first chemotherapy course.

### Statistical considerations

Correlations between AUC_MTX_ and the other variables were analysed by the Spearman test. The impact of studied variables on severe toxicity and complete response rate was analysed by logistic regression. Severe toxicity was defined by the onset of one of two major events: toxic death or interruption of chemotherapy due to toxicity. Complete response was defined as the disappearance of all evidence of lymphoma.

CL_crea_, AUC_MTX_, DI_MTX_, and IR_MTX_ were firstly analysed as continuous variables; then, quartiles values were applied as cutoff to differentiate the risk groups (categorical variables): lower quartile for CL_crea_ (85 ml min^−1^) and upper quartile for DI_MTX_ (1000 mg m^−2^ wk^−1^), for AUC_MTX_ (1100 *μ*mol h l^−1^), and for IR_MTX_ (800 mg m^−2^ h^−1^).

Survival curves were generated by the Kaplan–Meier method. The overall survival (OS) was calculated from diagnosis to the date of death or the last date of follow-up. Impact on survival of clinical and therapeutic variables was evaluated through the log-rank test. The independent prognostic value of variables was analysed using Cox proportional hazard model. All the probability values were two-sided. All the analyses were carried out using the Statistica 4.0 statistical package for Windows (Statsoft Inc, 1993, Tulsa, OK 74104, USA).

## RESULTS

### Study group

The study group consisted of 45 patients treated between 1995 and 2001 (Calderoni and Aebi 2002; Pasini *et al*. 2002; Ferreri *et al*. 2002a). Patients’ characteristics and extent of disease at diagnosis are summarised in Table 1

**Table 1:** Patients’ characteristics and extension of disease at diagnosis

	**Entire series**
No.	45
Median age (range)	54 (25–76)
>70 years	2 (4%)
Males	29 (64%)
	
*Performance status (ECOG score)*	
0–1	19 (42%)
2	13 (29%)
3	11 (24%)
4	2 (4%)
Prior cancer^a^	2 (4%)
	
*Histotype (REAL/WHO Classification)*	
Diffuse large B-cell lymphoma	42 (93%)
Anaplastic large-cell Ki1 lymphoma	1 (2%)
Unclassified	2 (4%)
High LDH serum level^b^	13/38 (34%)
Intraocular disease^b^	1/40 (3%)
Positive CSF cytology examination^b^	2/27 (7%)
Elevated CSF protein levels^b^,^c^	13/19 (68%)
Multiple lesions^b^	25/45 (56%)
Involvement of deep structures^b^,^d^	25/44 (57%)

CSF=cerebrospinal fluid.

a Prior cancers: renal cell carcinoma and Waldenstrom's macroglobulinaemia.

b Ratio between the number of positive cases and the number of assessed patients.

c The cutoff to define normal CSF protein levels was 45 mg dl^−1^ in patients ⩽60 years and 60 mg dl^−1^ in patients >60 years.

d Deep structures of the brain: basal ganglia, corpus callosum, brain stem, and cerebellum. ECOG=Eastern Cooperative Oncology Group; LDH=Lactic Dehydrogenase; REAL=Reversed European American lymphoma.

. Chemotherapy regimens and MTX administration schedules are reported in Table 2

**Table 2:** Chemotherapy regimens

**Regimen**	**No. of patients**	**MTX dose (mg m^−2^)**	**MTX infusion (h)**	**MTX dose day**	**Other drugs**	**i.t. CHT**	**Planned no. of courses**	**Courses every weeks**
MTX alone	10	1000–3000	4	1 and 7	—	Yes/no	2–3	4
(Calderoni and Aebi, 2002)		8000	24					
MATILDE	11	3500	3^a^	1	AraC 2 g m^−2^ × 2 d 2	No	3	4
(Ferreri *et al*, 2002a)					IDA 15 mg m^−2^ × d 1			
					TTP 25 mg m^−2^ × d 3			
MTX+AraC	24	1000–2000^b^	24	1	AraC 2 g m^−2^ × 2 d 2–3	no	3	3
(Pasini *et al*, 2002)								

MTX = methotrexate; i.t. CHT = intrathecal chemotherapy; AraC = cytarabine; IDA = idarubicin; TTP = thiotepa.

a Infusion preceded by an initial MTX bolus.

b Four patients received 3500–8000 mg m^−2^ in 24-h infusion. DI_MTX_ (mean±s.d.) of the used chemotherapy regimens were 1638±1642, 844±180, and 780±1080 mg m^−2^ week^−1^ (*P*=0.01), respectively, for HD-MTX alone, MATILDE, and MTX+Ara-C combination.

. All patients were treated with adequate pre-MTX hydration, urinary alkalinisation, and escalated leucovorin dosages according to MTX serum levels. Dehydration, aciduria, renal or cardiac dysfunction, pleural effusion, or gastrointestinal tract obstruction were excluded before commencing treatment in all cases. Post-chemotherapy RT, which consisted of whole-brain irradiation, followed or not followed by a tumour-bed boost, was planned in all cases, but it was in fact performed as part of the first-line therapy in 31 patients, with median brain and tumour-bed doses of 36±5 and 42±9 Gy, and as part of salvage therapy in six cases. Anticonvulsants were administered in 31 patients (69%), and consisted of phenobarbital (100–150 mg d^−1^) in 27 cases, hydantoin (100–300 mg d^−1^) in two cases and carbamazepine (400–800 mg d^−1^) in two cases.

### MTX parameters

The mean value±s.d. of CL_crea_ was 119±57 ml min^−1^. The mean value±s.d. of AUC_MTX_ was 731±525 *μ*mol h l^−1^. Patients ⩽60 years old displayed a faster CL_crea_ (mean±s.d.: 118±38 *vs* 94±28 ml min^−1^; *P*=0.01), and a higher AUC_MTX_ (846±562 *vs* 502±359 *μ*mol h l^−1^; *P*=0.02) with respect to patients >60. According to the PS, patients with an ECOG score of 3–4 displayed a similar CL_crea_ (100±28 *vs* 112±35 ml min^−1^; *P*=0.17) and a similar AUC_MTX_ (1397±1208 *vs* 1238±836 *μ*mol h l^−1^; *P*=0.53) with respect to patients with a PS of 0–2. The mean value±s.d. of DI_MTX_ was 992±1140 mg m^−2^ week^−1^. Seven patients (16%) received only one course of chemotherapy (severe toxicity in five, no response in two), 16 (36%) received two, 14 (31%) received three, and eight (18%) received more than three courses. No difference in the used MTX dose according to age or PS was observed; the proportion of patients ⩽60 years old and >60 treated with a dose >3 g m^−2^ was similar (47 *vs* 47%, *P*=0.99); 53% of patients with PS 0–2 and 31% of patients with PS 3–4 received an MTX dose >3 gm^−2^ (*P*=0.19). No cases of reduction of more than 25% of the MTX dose in further courses with respect to the planned dose were observed. The MTX dose of the further courses was increased by more than 25% with respect to the MTX dose of the first course in three (7%) cases. The mean value±s.d. of IR_MTX_ during the first chemotherapy course was 475±423 mg m^−2^ h. This parameter remained unmodified during further courses in all cases.

### AUC_MTX_-determining variables

Variables significantly correlated with the AUC_MTX_ are reported in Table 3

**Table 3:** Correlations between AUC_MTX_ and the other analysed variables

**Variables**	**Subgroups**	**No.**	**AUC_MTX_ (mean±s.d. *μ*mol h l^−1^)**	***P***
Age	⩽60 years	30	846±562	
	>60 years	15	502±359	0.02
				
Sex	Females	16	540±398	
	Males	29	837±562	0.08
				
PS	0–1	19	927±614	
	2–4	26	589±405	0.08
				
Anticonvulsants	No	14	1028±662	
	Yes	31	598±394	0.04
				
IR_MTX_ (mg m^−2^ h^−1^)	⩽800	33	635±564	
	>800	12	996±274	0.003
				
DI_MTX_ (mg m^−2^ week^−1^)	⩽1000	33	559±392	
	>1000	12	1206±567	0.0003
				
CL_crea_ (ml min^−1^)	⩽85	12	604±434	
	>85	33	778±553	0.35
				
				
Chemotherapy regimen	HD-MTX	10	857±658	
	MATILde	11	1043±240	
	MTX+AraC	24	536±491	0.008

PS=performance status according to the ECOG score.

. Anticonvulsant use and age correlated inversely with AUC_MTX_, while a direct correlation between AUC_MTX_ and IR_MTX_ and DI_MTX_ was observed. No correlation with sex, performance status (PS), and CL_crea_ was observed. Patients treated with MATILde chemotherapy regimen achieved significantly higher AUC_MTX_ values.

### Severe toxicity

The predictive value of MTX variables on severe toxicity was analysed considering toxic death (*n*=2) and interruption of chemotherapy due to toxicity (*n*=5) as events. Severe toxicity consisted of pulmonary thromboembolism in two cases (lethal in both), sepsis in two, acute renal failure in one, and persistent grade IV thrombocytopenia in two. These events were observed during the first two courses of chemotherapy. As reported in Table 4

**Table 4:** Logistic regression: variables correlated to severe toxicity (*n*=7) and complete remission rate (*n*=18) after primary chemotherapy

**Variables**	**Subgroups**	**No.**	**Severe toxicity**	***P***	**Complete response**	***P***
Age	⩽60 years	30	3 (10%)		11 (37%)	
	> 60 years	15	4 (27%)	0.28	7 (46%)	0.26
						
PS	0–1	16	2 (13%)		10 (62%)	
	2–4	29	5 (17%)	0.14*^α^*	8 (28%)	0.19
						
Anticonvulsants	No	14	1 (7%)		6 (43%)	
	Yes	31	6 (19%)	0.97	12 (39%)	0.51
						
						
IR_MTX_ (mg m^−2^ h^−1^)	⩽800	33	4 (12%)		15 (45%)	
	>800	12	3 (33%)	0.11	3 (25%)	0.49
						
DI_MTX_ (mg m^−2^ week^−1^)	⩽1000	33	6 (18%)		14 (42%)	
	>1000	12	1 (8%)	0.48	4 (33%)	0.52
						
CL_crea_ (ml min^−1^)	⩽85	12	4 (33%)		8 (67%)	
	>85	33	3 (9%)	0.05	10 (30%)	0.02
						
AUC_MTX_ (*μ*mol h l^−1^)	⩽1100	34	6 (17%)		14 (41%)	
	>1100	11	1 (9%)	0.45	4 (36%)	0.95
						
Chemotherapy regimen	HD-MTX	10	0 (0%)		2 (20%)	
	MATILde	11	3 (27%)		3 (27%)	
	MTX+AraC	24	4 (17%)	0.25	13 (54%)	0.08

HD-MTX=high-dose methotrexate. ^a^The incidence of severe complications was significantly higher in patients with a PS>2 with respect to the others (41 *vs* 9%, *P*=0.002). An additional logit analysis with patients grouped according to PS⩽2 *vs* >2 confirmed the independent association between toxicity and CL_crea_.

, CL_crea_ was independently associated with severe toxicity; a significantly higher toxicity rate was observed in patients with a CL_crea_ ⩽85 ml min^−1^. Importantly, a DI_MTX_ >1000 mg m^−2^/week, and an AUC_MTX_ >1100 *μ*mol h l^−1^ were not related to a higher toxicity.

### Objective response

After primary chemotherapy, 18 patients (40%) achieved a complete remission and 16 (36%) a partial response (overall response rate= 76%); four patients (9%) had stable disease, five (11%) experienced progressive disease, and two (4%) died of toxicity. As reported in Table 4, a slow CL_crea_ (⩽85 ml min^−1^) was significantly and independently associated with a higher complete remission rate.

### Overall survival

A total of 26 patients experienced failure: early progression of the disease in nine cases, relapse after initial response (complete and partial) in 15 and toxic death in two cases, with a 2-year failure-free survival of 50±8%. In all, 22 patients are alive (19 NED) at a median follow-up of 31 months (range 4–72 months), with a 3-year OS of 40±9%. The cause of death was lymphoma in 20 cases, acute toxicity in two, and unrelated disorder in one.

**Figure 1:**
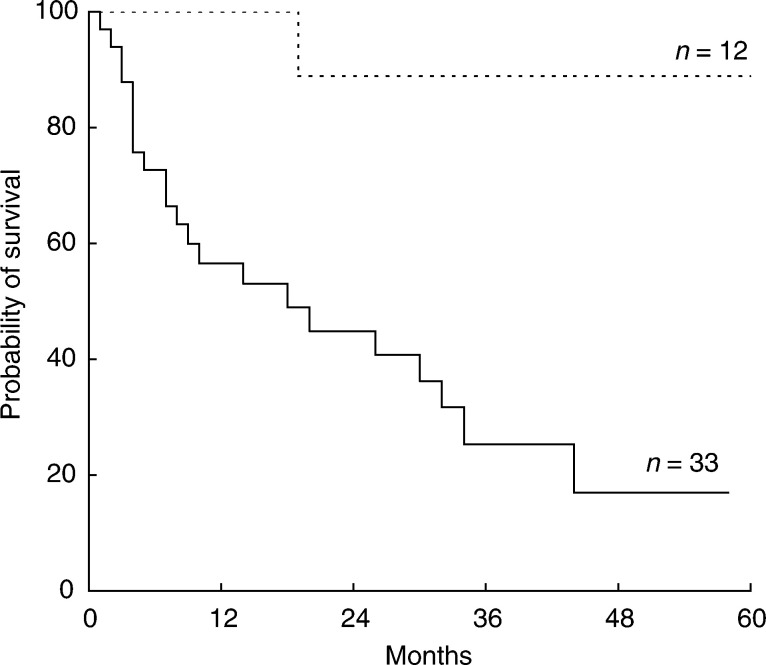
OS curves for patients grouped according to the CL_crea_. Patients with a slow CL_crea_ (⩽85 ml min^−1^; dotted line) showed a better OS with respect to patients with a fast CL_crea_ (>85 ml min^−1^; continued line).

**Figure 2:**
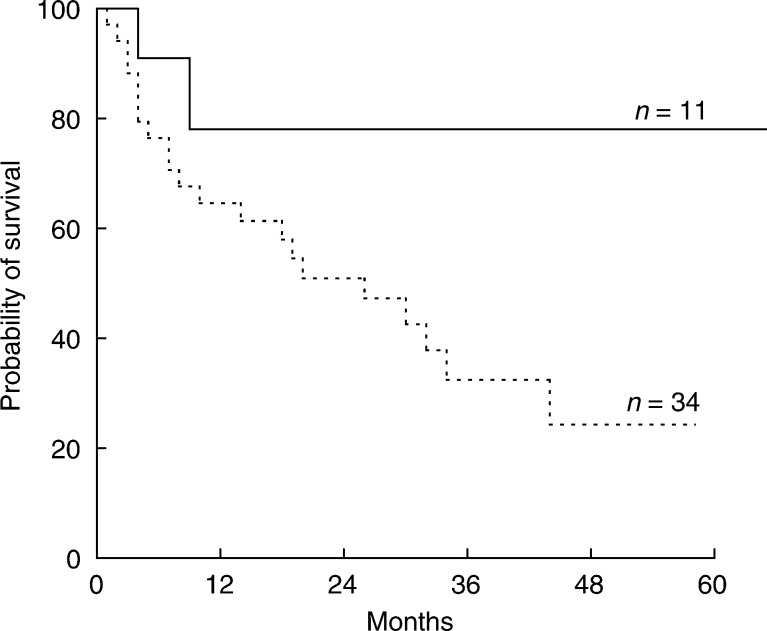
OS curves for patients grouped according to the AUC_MTX_. Patients treated with an AUC_MTX_ >1100 *μ*mol h  l^−1^ (continued line) showed a significantly better survival with respect to those treated with an AUC_MTX_ ⩽1100 *μ*mol h  l^−1^ (dotted line).

Univariate analyses showed that patients with a slow CL_crea_ (⩽85 ml min^−1^) survived longer than patients with a fast CL_crea_ (>85 ml min^−1^), with 3-year OS values of 88±13 and 25±9% (*P*=0.0005), respectively (Figure 1

). Patients treated with an AUC_MTX_ >1100 *μ*mol h l^−1^ survived significantly longer than patients treated with lower levels (3-year OS: 78±12 *vs* 32±9%; *P*=0.05) (Figure 2

). The use of anticonvulsants and IR_MTX_ was not associated with survival, and no significant difference in the efficacy of the used chemotherapy regimens was observed. Multivariate analysis (Table 5

**Table 5:** Multivariate analysis: impact on overall survival of studied variables

**Variables**	**Subgroups**		**Odds ratio (CI 95%)**	***P***
Age	Continuous variable		1.06 (1.01–1.11)	0.04
PS	0–2	3–4	2.55 (1.92–7.02)	0.05
Anticonvulsant	No	Yes	1.46 (0.28–7.53)	0.65
IR_MTX_ (mg m^−2^ h^−1^)	⩽800	>800	0.51 (0.11–2.49)	0.41
DI_MTX_ (mg m^−2^ week^−1^)	⩽1000	>1000	3.38 (0.53–4.98)	0.21
CL_crea_ (ml min^−1^)	⩽85	>85	6.01 (3.04–9.77)	0.005
AUC_MTX_ (*μ*mol h l^−1^)	⩽1100	>1100	0.11 (0.01–0.77)	0.03
Cytarabine^a^	No	Yes	1.19 (0.34–4.11)	0.78
Alkylating agents^a^	No	yes	4.41 (0.75–5.61)	0.16

a Similar results were obtained when analysis was performed according to the chemotherapy regimen.

) confirmed the independent prognostic value of CL_crea_ and AUC_MTX_.

## DISCUSSION

The present study focused on the impact on toxicity and outcomes of CL_crea_, AUC_MTX_, DI_MTX_, and IR_MTX_ in a multicentre retrospective series of 45 immunocompetent patients with PCNSL. This series is representative of PCNSL patients currently treated with HD-MTX-based chemotherapy, since it displays similar median age, PS distribution, histotypes, and ocular and meningeal infiltration rates with respect to more comprehensive unselected retrospective series (Ferreri *et al*, 2002b), and to the largest published prospective trials (O’Brien *et al*, 2000; Deangelis *et al*, 2002). A clear relationship between the studied variables and therapeutic outcome is difficult to establish, considering the multitude of other factors, such as protein binding, membrane transport, dihydrofolate reductase levels, tissue distribution, or concurrent drugs, which may also influence the efficacy of MTX. Nevertheless, as has been reported for other malignancies (Evans *et al*, 1986; Graf *et al*, 1994; Delepine *et al*, 1995; Bacci *et al*, 1998), the characterisation of the MTX variables investigated could be useful to identify different risk groups and to define the optimal administration schedule of this drug in PCNSL patients.

HD-MTX is the most effective drug against PCNSL; any regimen without this drug is associated with outcomes which are no better than with RT alone (Schultz *et al*, 1996; O’Neill *et al*, 1999; Mead *et al*, 2000). When used as primary treatment, alone or combined with other drugs, followed or not followed by RT, HD-MTX produces a response rate of 70–80%, with a 2-year OS of 60–70% (Ferreri *et al*, 2000). The survival benefit of the addition of other drugs to HD-MTX is matter of debate, considering that not only do randomised trials comparing mono-chemotherapy with HD-MTX and poly-chemotherapy not exist, but also that the activity of these drugs has not been assessed as a single drug in prospective trials. Thus, HD-MTX remains a crucial drug against PCNSL, being an irreplaceable component of primary chemotherapy. Nevertheless, the prognostic role of the AUC_MTX_ and DI_MTX_ as well as the optimal MTX dose, IR and dose timing of this drug have not been clearly defined in PCNSL. A single study comparing some MTX parameters in PCNSL patients treated with blood–brain barrier disruption or with systemic chemotherapy has been reported (Zylber-Katz *et al*, 2000), but their impact on outcome has not been analysed. Conversely, the prognostic role of MTX pharmacokinetics has been reported in other malignancies in which this drug plays a critical role, such as acute leukaemia (Evans *et al*, 1986) and osteosarcoma (Graf *et al*, 1994; Delepine *et al*, 1995; Bacci *et al*, 1998). Patients with acute leukaemia have been grouped according to a slow, medium or fast CL_MTX_, obtaining an inverse association with outcome (Evans *et al*, 1986). A significant correlation between a faster CL_MTX_ and lower serum and cerebrospinal fluid (CSF) MTX concentrations has also been documented, suggesting an insufficient treatment both of the brain and meninges, and a greater risk of CNS relapse in this subgroup of leukaemia patients (Evans *et al*, 1983). Likewise, a significant survival effect of serum peak concentration of MTX has been reported in osteosarcoma (Graf *et al*, 1994).

Our study suggests that CL_crea_ and AUC_MTX_ are independent predictors of MTX efficacy in PCNSL patients also. A CL_crea_ ⩽85 ml min^−1^ was associated with a higher complete remission rate and better survival, which was independent of age, PS, DI_MTX_, IR_MTX_, and other therapeutic variables. Patients treated with an AUC_MTX_ >1100 *μ*mol h l^−1^ showed a significantly better survival with respect to those treated with lower AUC_MTX_ levels. CL_MTX_ is defined based upon CL_crea_; decreased CL_crea_ represents decreased CL_MTX_, which for a given dose would produce an increased AUC_MTX_. However, in the present series, CL_crea_ and AUC_MTX_ are two independent variables, which is explained by the heterogeneity in MTX dose, DI_MTX_, and IR_MTX_, as well as by differences in the MTX metabolism, according to the drug infusion duration (see below). A strongly inverse correlation between CL_crea_ and AUC_MTX_ could be observed, for example, in a prospective trial, where the used MTX dose and schedule is the same for the entire series. Considering that AUC_MTX_ is significantly correlated, among others, with DI_MTX_, IR_MTX_, and anticonvulsant therapy, changes in these parameters could lead to significant changes in AUC_MTX_ and efficacy. Importantly, as reported in Table 4, a higher AUC_MTX_ was not associated with a higher incidence of severe toxicity (9 *vs* 17%, *P*=0.45). The single case of severe toxicity in the group of patients treated with an AUC_MTX_ >1100 *μ*mol h l^−1^ consisted of nonlethal persistent thrombocytopenia, without bleeding complications in a patient with a CL_crea_ of 186 ml min^−1^. On the other hand, four of the five cases of severe renal and haematological toxicity were observed in patients with slow CL_crea_. A close follow-up with haematologic profile and renal function assessment appears advisable in this subgroup of patients.

Pretreatment CL_crea_ assessment could also be useful to identify groups of PCNSL patients with different MTX efficacy, and the use of higher doses in patients with a fast CL_crea_ should be critically considered. The choice of the MTX dose is a relevant issue in PCNSL, especially because of the high interpatient and intrapatient variability of MTX pharmacokinetics. For example, in a recently published trial (Batchelor *et al*, 2003), the MTX dose (8 g m^−2^) was adjusted based on the pre-chemotherapy CL_crea_. The dose was reduced by the percentage decrease of this variable below 100 ml min^−1^. The schedule employed was well tolerated; however, a detailed analysis of the impact on activity and toxicity of this strategy was not provided. Our data suggest that changes in MTX dose according to the pretreatment CL_crea_ could lead to more suitable AUC_MTX_ levels, with a consequent efficacy improvement. This interesting hypothesis will be studied in a prospective series treated with a homogeneous MTX schedule.

DI is a debated outcome-conditioning factor in aggressive systemic lymphomas, while its role has not been investigated in PCNSL patients treated with conventional strategies. In the present series, DI_MTX_ was not associated with survival or toxicity. However, the significant association between a DI_MTX_ >1000 mg m^−2^ week^−1^ and higher AUC_MTX_ levels seems to suggest that, when administered every 3–4 weeks, a MTX dose ⩾3000 mg m^−2^ could produce better results than lower doses. This also seems to be supported by the MSKCC and RTOG experience (Deangelis *et al*, 1992, 2002). In a previous trial (Deangelis *et al*, 1992), MTX administered at a dose of 1 g m^−2^ produced an overall response rate of 64% and a 5-year OS of 28%, while, in a recently reported trial (Deangelis *et al*, 2002), the use of a 3.5-g m^−2^ MTX dose produced an overall response rate of 90% and a 5-year OS of 50%. Analysed together, these data suggest that a higher amount of MTX administered in a single dose increases drug exposure and activity. Moreover, as previously reported (Pitman and Frei, 1977; Evans *et al*, 1983), this strategy seems to lead to a higher diffusion of the drug across the blood–brain barrier and increased drug concentrations in the CSF, with a potential positive impact on efficacy against PCNSL.

From the present analysis, no significant association between IR_MTX_ and survival was observed. This could be due to the strong correlation observed between this parameter and AUC_MTX_. The identification of the best IR_MTX_ in PCNSL needs further studies. In the meantime, an IR_MTX_ of >800 mg m^−2^ h^−1^ appears advisable, since it is associated with higher AUC_MTX_ and does not display significantly higher toxicity in comparison to slower rates.

Anticonvulsants are commonly used in PCNSL patients presenting seizures. These drugs interact with hepatic aldehyde oxidase, which constitutes a major mechanism for MTX degradation (Jacobs *et al*, 1976). Anticonvulsant therapy increases the systemic CL_MTX_, as well as the level of other cytostatics, and is associated with lower efficacy of chemotherapy in children treated for acute lymphoblastic leukaemia (Relling *et al*, 2000). This effect seems to be more intense in patients treated with HD-MTX by a 24-h infusion, in whom about 40% of the drug is metabolised in the liver, whereas, when HD-MTX is administered as a short intravenous infusion (4–6 h), most of the drug is cleared by the kidneys (Evans *et al*, 1983). In the present analysis, no association between anticonvulsant use and toxicity and outcome was observed, and the impact of these drugs in patients treated with a 24-h infusion cannot be analysed, considering that all these patients received anticonvulsant therapy. The hypothesis that MTX dose adjustments are needed when anticonvulsants are contemporarily used should be better explored.

To identify new active drugs and combinations remains the most important strategy to improve therapeutic results in PCNSL patients, and any effort to define the best administration schedule for HD-MTX should be encouraged. With certain limitations due to their retrospective nature, our data seem to suggest that slow CL_crea_ and high AUC_MTX_ are independently associated with better outcome in PCNSL patients. Comprehensively, these interesting findings deserve to be assessed in prospective trials. In the meantime, a MTX dose ⩾3000 mg m^−2^ administered in a 4- or 6-h infusion, every 3–4 weeks, appears an advisable schedule to adopt in clinical practice. The need to increase the MTX dose to ensure adequate exposure, such as higher AUC_MTX_ values, in patients with a fast CL_crea_, should be critically considered.

## References

[bib1] Bacci G, Ferrari S, Delepine N, Bertoni F, Picci P, Mercuri M, Bacchini P, Brach dP, Tienghi A, Comandone A, Campanacci M (1998) Predictive factors of histologic response to primary chemotherapy in osteosarcoma of the extremity: study of 272 patients preoperatively treated with high-dose methotrexate, doxorubicin, and cisplatin. J Clin Oncol 16: 658–663 9469355 10.1200/JCO.1998.16.2.658

[bib2] Batchelor T, Carson K, O’Neill A, Grossman SA, Alavi J, New P, Hochberg F, Priet R (2003) Treatment of primary CNS lymphoma with methotrexate and deferred radiotherapy: a report of NABTT 96-07. J Clin Oncol 21: 1044–1049 12637469 10.1200/JCO.2003.03.036

[bib3] Calderoni A, Aebi S (2002) Combination chemotherapy with high-dose methotrexate and cytarabine with or without brain irradiation for primary central nervous system lymphomas. J Neurooncol 59: 227–230 12241119 10.1023/a:1019993018162

[bib4] Cockcroft DW, Gault MH (1976) Prediction of creatinine clearance from serum creatinine. Nephron 16: 31–41 1244564 10.1159/000180580

[bib5] Deangelis LM, Seiferheld W, Schold SC, Fisher B, Schultz CJ (2002) Combination chemotherapy and radiotherapy for primary central nervous system lymphoma: Radiation Therapy Oncology Group Study 93-10. J Clin Oncol 20: 4643–4648 12488408 10.1200/JCO.2002.11.013

[bib6] Deangelis LM, Yahalom J, Thaler HT, Kher U (1992) Combined modality therapy for primary CNS lymphoma. J Clin Oncol 10: 635–643 1548527 10.1200/JCO.1992.10.4.635

[bib7] Delepine N, Delepine G, Cornille H, Brion F, Arnaud P, Desbois JC (1995) Dose escalation with pharmacokinetics monitoring in methotrexate chemotherapy of osteosarcoma. Anticancer Res 15: 489–494 7763028

[bib8] Evans WE, Crom WR, Abromowitch M, Dodge R, Look AT, Bowman WP, George SL, Pui CH (1986) Clinical pharmacodynamics of high-dose methotrexate in acute lymphocytic leukemia. Identification of a relation between concentration and effect. N Engl J Med 314: 471–477 3456079 10.1056/NEJM198602203140803

[bib9] Evans WE, Hutson PR, Stewart CF, Cairnes DA, Bowman WP, Rivera G, Crom WR (1983) Methotrexate cerebrospinal fluid and serum concentrations after intermediate-dose methotrexate infusion. Clin Pharmacol Ther 33: 301–307 6600662 10.1038/clpt.1983.37

[bib10] Ferreri AJM, Bernardi M, Dell’Oro S, Brandes AA, Reni M, Ciceri F, Pasetto LM, Spina M, Stelitano C, Balzarotti M, Illariuci F, Ponzoni M, Franzin A, Danesi R, Villa E (2002a) CLIMT-2: an ongoing phase-II multicentre trial of MATILde chemotherapy regimen (methotrexate–cytarabine–thiotepa–idarubicin) in HIV-negative Primary CNS Lymphomas (PCNSL). Proc Annu Meet Am Soc Clin Oncol 21: 267a (abstract: 2077)

[bib11] Ferreri AJM, Reni M, Pasini F, Calderoni A, Tirelli U, Pivnik A, Aondio GM, Ferrarese F, Gomez H, Ponzoni M, Borisch B, Berger F, Chassagne C, Iuzzolino P, Carbone A, Weis J, Pedrinis E, Motta T, Jouvet A, Barbui T, Cavalli F, Blay JY (2002b) A multicenter study of treatment of primary CNS lymphoma. Neurology 58: 1513–1520 12034789 10.1212/wnl.58.10.1513

[bib12] Ferreri AJM, Reni M, Villa E (2000) Therapeutic management of primary central nervous system lymphoma: lessons from prospective trials. Ann Oncol 11: 927–937 11038028 10.1023/a:1008376412784

[bib13] Graf N, Winkler K, Betlemovic M, Fuchs N, Bode U (1994) Methotrexate pharmacokinetics and prognosis in osteosarcoma. J Clin Oncol 12: 1443–1451 8021736 10.1200/JCO.1994.12.7.1443

[bib14] Hryniuk WM, Goodyear M (1990) The calculation of received dose intensity. J Clin Oncol 8: 1935–1937 2230885 10.1200/JCO.1990.8.12.1935

[bib15] Jacobs SA, Stoller RG, Chabner BA, Johns DG (1976) 7-Hydroxymethotrexate as a urinary metabolite in human subjects and rhesus monkeys receiving high dose methotrexate. J Clin Invest 57: 534–538 1062383 10.1172/JCI108308 PMC436681

[bib16] Mead GM, Bleehen NM, Gregor A, Bullimore J, Shirley D, Rampling RP, Trevor J, Glaser MG, Lantos P, Ironside JW, Moss TH, Brada M, Whaley JB, Stenning SP (2000) A medical research council randomized trial in patients with primary cerebral non-Hodgkin lymphoma: cerebral radiotherapy with and without cyclophosphamide, doxorubicin, vincristine, and prednisone chemotherapy. Cancer 89: 1359–1370 11002232

[bib17] O’Brien P, Roos D, Pratt G, Liew K, Barton M, Poulsen M, Olver I, Trotter G (2000) Phase II multicenter study of brief single-agent methotrexate followed by irradiation in primary CNS lymphoma. J Clin Oncol 18: 519–526 10653867 10.1200/JCO.2000.18.3.519

[bib18] O’Neill BP, Wang CH, O'Fallon JR, Colgan JD, Earle JD, Krigel RL, Brown LD, McGinnis WL (1999) Primary central nervous system non-Hodgkin's lymphoma (PCNSL): survival advantages with combined initial therapy? A final report of the North Central Cancer Treatment Group (NCCTG) Study 86-72-52. Int J Radiat Oncol Biol Phys 43: 559–563 10078637 10.1016/s0360-3016(98)00450-7

[bib19] Pasini F, Todeschini G, Ambrosetti A, Nicolato A, Miseria S, Durante E, Zaninelli M, Manno P, Tecchio C, Pizzolo G, Cetto GL (2002) A phase II study of high-dose (HD) methotrexate and HD cytarabine followed by radiotherapy in primary CNS lymphomas (PCNSL). Ann Oncol 13(Suppl. 2): 79 (Abstract 266)12078908

[bib20] Pitman SW, Frei E (1977) Weekly methotrexate-calcium leucovorin rescue: effect of alkalinization on nephrotoxicity; pharmacokinetics in the CNS; and use in CNS non-Hodgkin's lymphoma. Cancer Treat Rep 61: 695–701 18282

[bib21] Relling MV, Pui CH, Sandlund JT, Rivera GK, Hancock ML, Boyett JM, Schuetz EG, Evans WE (2000) Adverse effect of anticonvulsants on efficacy of chemotherapy for acute lymphoblastic leukaemia. Lancet 356: 285–290 11071183 10.1016/S0140-6736(00)02503-4

[bib22] Reni M, Ferreri AJ, Garancini MP, Villa E (1997) Therapeutic management of primary central nervous system lymphoma in immunocompetent patients: results of a critical review of the literature. Ann Oncol 8: 227–234 9137790 10.1023/a:1008201717089

[bib23] Reni M, Ferreri AJ, Guha-Thakurta N, Blay JY, Dell'Oro S, Biron P, Hochberg FH (2001) Clinical relevance of consolidation radiotherapy and other main therapeutic issues in primary central nervous system lymphomas treated with upfront high-dose methotrexate. Int J Radiat Oncol Biol Phys 51: 419–425 11567816 10.1016/s0360-3016(01)01639-x

[bib24] Schultz C, Scott C, Sherman W, Donahue B, Fields J, Murray K, Fisher B, Abrams R, Meis-Kindblom J (1996) Preirradiation chemotherapy with cyclophosphamide, doxorubicin, vincristine, and dexamethasone for primary CNS lymphomas: initial report of radiation therapy oncology group protocol 88-06. J Clin Oncol 14: 556–564 8636771 10.1200/JCO.1996.14.2.556

[bib25] Zylber-Katz E, Gomori JM, Schwartz A, Lossos A, Bokstein F, Siegal T (2000) Pharmacokinetics of methotrexate in cerebrospinal fluid and serum after osmotic blood–brain barrier disruption in patients with brain lymphoma. Clin Pharmacol Ther 67: 631–641 10872645 10.1067/mcp.2000.106932

